# The promising performance of manganese gluconate as a liquid redox sulfur recovery agent against oxidative degradation

**DOI:** 10.1016/j.heliyon.2021.e06743

**Published:** 2021-04-15

**Authors:** Andreas Widodo, Yestria Yaswari, Rina Mariyana, Aditya Farhan Arif, Tirto Prakoso, Tri Partono Adhi, Tatang Hernas Soerawidjaja, Ronny Purwadi, Antonius Indarto

**Affiliations:** a PT. Energy Management Indonesia (EMI), Jl. Pancoran Indah I No.52, Jakarta, Indonesia; b PT. Rekayasa Industri (REKIND), Jl. Kalibata Timur I no 36, Jakarta, Indonesia; c Department of Chemical Engineering, Institut Teknologi Bandung, Labtek X, Kampus ITB, Jalan Ganesha 10, Bandung, 40132, Indonesia; d Department of Bioenergy Engineering and Chemurgy, Institut Teknologi Bandung, Jalan Let. Jen. Purn. Dr. (HC), Mashudi No. 1, Sumedang, Indonesia; e Department of Food Engineering, Institut Teknologi Bandung, Jalan Let. Jen. Purn. Dr. (HC), Mashudi No. 1, Sumedang, Indonesia

**Keywords:** Manganese gluconate, Sulfur recovery, Natural gas, LRSR, NTA, EDTA, Degradation

## Abstract

This work studied the oxidative degradation performance of manganese gluconate as a liquid redox sulfur recovery (LRSR) agent. The degradation of gluconate in an aerated sulfide containing 0.1 M manganese/0.8 M gluconate/pH 13 solution was 11% in 47 h and 20% in 100 h of reaction time. With the total price of chelates being more or less comparable, these were superior to the degradation resistance of EDTA chelate in a solution of 0.1 M iron/0.2 M EDTA/pH 8 which degraded by about 30% in 47 h, and NTA in Fe-NTA (0.1 M metal/0.2 M chelate/pH 6.5), which was degraded by 40% in 100 h of reaction time. At pH of 13, 0.1 M Metal, and 0.8 M gluconate, manganese degraded gluconate more severely than iron and copper. At a lower chelate to metal molar ratio (RCM) of 2 and as well as at a lower pH of 10, the manganese gluconate degradation, expressed as relative concentration to its initial concentration, was faster than at RCM of 8 and pH of 13. All of these observations can be explained among others by the well-known Fenton reaction hydroxyl radicals mechanism as the main cause of the degradation process.

## 1 Introduction

Natural gas is widely utilized in various industries as an energy source or raw material. Impurities like CO_2_ and H_2_S must be removed from sour gas such that the gas meets sweet gas quality specifications and transportation requirements. To protect people and the environment H_2_S must also be recovered as safer substances which then can hopefully be utilized for other applications. A vast number of commercial sulfur recovery technologies are available for natural gas treating applications. One of the technologies which are often suitable for 1–20 metric ton sulfur per day is the iron chelate Liquid Redox Sulfur Recovery (LRSR) process [1, 2]. The technology offers several advantages. For this scale, it is more economical than the classical gas-phase indirect oxidation Claus process. This would be particularly true for acid gas with low H_2_S/CO_2_ molar ratio (1–3%), as it does not need an acid gas enrichment unit. Compared to the probably somewhat less expensive direct gas-phase oxidation technology LRSR can give considerably higher sulfur recovery efficiency [1]. Here, LRSR also has better tolerance towards heavy hydrocarbons, BTX, and mercaptans. Despite all of these advantages, the use of LRSR technology was economically restricted to low sulfur capacity due to two major limitations: (1) high solution circulation rate and (2) high chemical make-up due to rapid oxidative degradation of chelates.

Several investigations have been carried out to overcome or reduce degradation problems [3, 4]. One of which was our work that suggested manganese gluconate could be a potential, greener, more economical, and stable redox agent for the LRSR process [5]. However as this is still a novel concept, to our knowledge no information on manganese gluconate degradation in LRSR systems has been reported in the open literature. Preliminary observations were made in our previous work, but still preliminary and qualitative. Hence it needs to be confirmed by quantitative data. In contrast, considerable detailed studies on degradation and degradation mechanisms of conventional chelating agents (ie., NTA and EDTA) are already available in the literature [3, 6, 7]. Chen et al. investigated iron (0.018M) - NTA (0.036M), and iron (0.018M) - EDTA (0.036M) system at 25 °C, with and without H_2_S gas feed [6]. At pH between 7 and 8.5, for instance, half of the initial NTA in an aerated LRSR iron chelate system with H_2_S gas feed had already degraded only within 25–40 h. However, there was no degradation product observed after two days when iron (III) complex was charged with sulfide without aeration or was aerated without sulfide. Based on this and other evidence they proposed a degradation mechanism with hydroxyl radicals produced by Fenton reaction as the main degrading agent. Piché measured Fe(III) EDTA degradation at pH 8 to 9, 25 °C–55 °C without sulfide and aeration [7]. Contrary to Chen et.al. experiments, he observed the average degradation rate per day of EDTA in iron (III) (0.45 mM) EDTA (0.9 mM) at pH 9 at 25 °C to be from 2.8% to 7.2%, depending on solution ionic strengths. Another distinction between the two reports was that degradation becomes more rapid at lower pH in Chen's case, while in Piché's case was the opposite. Sönmez reported that within 700 h the concentration of EDTA of Fe (0.1 M) -EDTA (0.15 M) solution at 30 °C and 60 °C went down by 10% and 50% of the initial concentration, respectively [3]. The solutions were aerated in the absence of sulfide. Degradation even took place when the solution was sparged with carbon dioxide rather than air. This was attributed to the internal electron transfer degradation mechanism or autocatalyzed oxidation. Here EDTA was oxidized by chelated Fe(III) directly rather than by hydroxyl radicals [8]. Thus concerning the extent of each chelate degradation mechanism, it may still look to be somewhat unclear.

As for gluconate, literature reported that this chelate can indeed be degraded by Fenton-like mechanism [9]. In addition, it can also be deliberately degraded (in this case, decarboxylated) by H_2_O_2_ in the presence Fe(III) acetate through the Ruff degradation mechanism [10]. As observed in iron chelate complexes, in the regeneration reaction of manganese complexes by oxygen, there is also a reaction that produces H_2_O_2_ [9]. Therefore, both manganese and iron gluconate complexes can undergo degradation. Among carbohydrate derivatives, oxidation of gluconic acid by iron (II) through the Fenton and Ruff reactions was a relatively slow reaction [10]. Valachova et al. reported that the addition of manganese inhibited the degradation of hyaluronan samples by the ascorbic Fenton catalyzed by iron or copper [11]. The inhibition has been attributed to the ability of manganese (II) to act as hydroxyl free radicals scavenger. Cheton and Archibald also reported the ability of some manganese complexes in reducing or blocking substance degradation caused by hydroxyl radicals generated by Fenton or similar mechanisms [12]. In one of the Cheton and Archibald experiments, Mn EDTA gave much lower degradation to 2 keto-4-thiomethylbutyrate (KMB) in hypoxanthine oxidase and ascorbate system than Fe-EDTA. The degradation resistance of iron gluconate at high temperatures which was lower than that of iron EDTA has been reported as well [13]. All of the previous information has been important motivations to further investigate the degradation resistance of manganese gluconate solution in the redox process for the LRSR process. Hence, this study was aimed to generate more quantitative data on the degradation of gluconate and factors that may influence it. Similar degradation experiments were also carried out for iron-NTA, iron-EDTA, iron gluconates, and copper gluconates for comparison and analysis purposes.

The total metal concentration of each complex tested was 0.1 M, with initial chelate concentrations were 0.2 M for NTA and EDTA, and around 0.8 M for gluconate. The concentrations of metal applied in these experiments was determined based on the high end of the typical range used in the commercial iron chelate process [14]. The chelate to metal molar ratio (RCM) and pH for each solution was selected based on metal solubility. When the work was carried out the price of commercial gluconate was around 25% of NTA and EDTA per mol. Therefore the concentration of the chelates was to some extend comparable price-wise for making performance comparison.

## 2 Experimental setup

### 2.1 Materials

Hydrated sodium sulfide (Na_2_S·X·H_2_O) was used as a source of sulfides (sulfur content of 34%) was purchased from Sigma Aldrich. The sulfur content in Na_2_S was determined by the iodometric titration method. NTA trisodium salt with a purity of 98%, EDTA tetrasodium salt with a purity of 97%, and technical sodium D-gluconate salt were used as chelates. NaOH (99% purity), MnCl_2_·4·H_2_O (99% purity), Fe_2_SO_4_, CuCl_2_ (analytical standard), and HCl were purchased from Merck, Germany. Manganese(II) Gluconate and Fe(II)gluconate (purity of >97%) were obtained from Jost Chemical, USA.

### 2.2 Solution preparation and reaction procedure

Fe(II)NTA and Fe(II)EDTA solution was respectively prepared by dissolving NTA trisodium salt/EDTA tetrasodium salt in nitrogen-purged double distilled water, to which ferrous sulfate was added to obtain an NTA/EDTA concentration of 0.2 M and 0.1 M of iron. Sodium hydroxide and/or HCl were then used to adjust pH to reach 6.5 and 8 respectively. Mn(II)gluconate solution was prepared by adding manganese gluconate or manganese chloride (MnCl_2_) to ca 0.8 M of nitrogen purged sodium gluconate solution until the manganese concentration reached 0.1 M. Sodium hydroxide was then further added to reach a pH of 13. Iron (II) gluconate and copper gluconate solutions were prepared by using ferrous gluconate or by adding iron sulfate (FeSO_4_) or CuCl_2_ to a 0.8 M sodium gluconate solution to reach a metal content of 0.1 M. Later, sodium hydroxide was then added to reach a pH of 13.

Complex solutions at 25 °C of Mn(II)-gluconate, Fe(II)-EDTA, and Fe(II)-NTA were then fed into the respective reactors. Air was bubbled into the reactor which contained 200 ml of solution, after previously being bubbled through 800 ml of distilled water to saturate with water. The aeration was carried out for several hours such that all metal had been oxidized to a higher oxidation state as indicated by color change. The volume of the liquid in the reactor was maintained with the addition of demineralized water. Sodium hydroxide was added during the reactions if necessary to maintain pH. Sodium sulfide, when used, was then fed twice a day to maintain an average feed rate of 5 mmol/L/h.

### 2.3 Chelate concentration measurement

To determine the rate of degradation, samples were taken from reactors and analyzed by a High Performance Liquid Chromatography (HPLC, Waters, isocratic pump 1515, autosampler 2707, RID 2414). The samples of NTA, EDTA, and gluconate were analyzed by HPLC using different treatments. The method taught by Parkes et al. was employed with necessary modifications for the measurement of NTA and EDTA [15]. For manganese gluconate, 1 ml of sample was added with HCl until the pH became 2 and the color changed from dark to clear solution. Afterward, the sample was diluted in 10 ml of distilled water and then filtered with a 0.22-micron filter to remove any deposits if present. In the case of Fe-NTA and Fe-EDTA samples, NaOH was added to 0.5 ml of the sample until it was transparent to precipitate all iron hydroxide and diluted until 10 ml. The precipitate was then separated with a 0.22-micron filter. A total of 5 ml of solution was then added with HCl to reach pH of 4. To the remaining solution, 12.5 mg of CuSO_4_ was added in 0.2 M NTA solution and 50 mg for 0.8 M NTA solution to form a Cu-NTA or Cu-EDTA complex. Thus the amount of mol CuSO_4_ added exceeded chelate initial molar quantity by ca. 50% to ensure all chelate was complexed. The sample was re-filtered with a 0.22-micron filter. Determination of NTA and EDTA concentrations was conducted by using HPLC (BondapakTM, C-18 column with a UV-Vis detector) at a maximum wavelength of 254 nm. The eluent was tetra-n-butyl ammonium hydroxide with a concentration of 0.4% and a pH of 7.5 with a flow rate of 1.5 mL/min. In the case of Gluconate, the analysis was conducted by using RI detector with Aminex HPX87H column. The eluent used was 5 mM H_2_SO_4_ solution with a flow rate of 0.6 mL/min with an operating temperature of 60 °C.

## 3 Results and discussions

### 3.1 Degradation of gluconate, NTA, and EDTA chelates without dissolved sulfides

**Figure 1:**
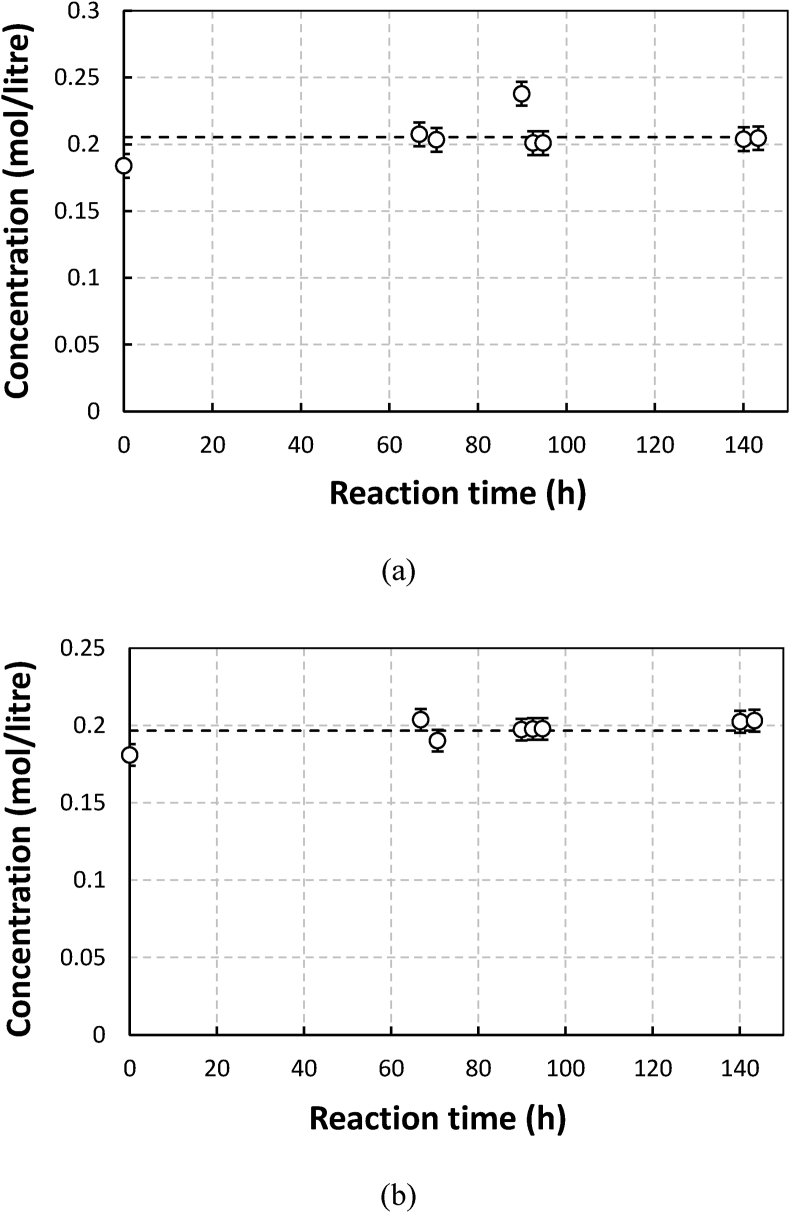
The stability of NTA ligands in an aerated NTA solution without sulfides at pH of 6.5: (a) without iron and (b) with iron (0.1 M).

Figure 1 shows concentrations of NTA and Fe-NTA solution at pH of 6.5 as a function of time when subjected to continuous aeration in the absence of sulfide. The initial concentration of NTA for both solutions was 0.2 M. The stability test was conducted for more than 100 h.

**Figure 2:**
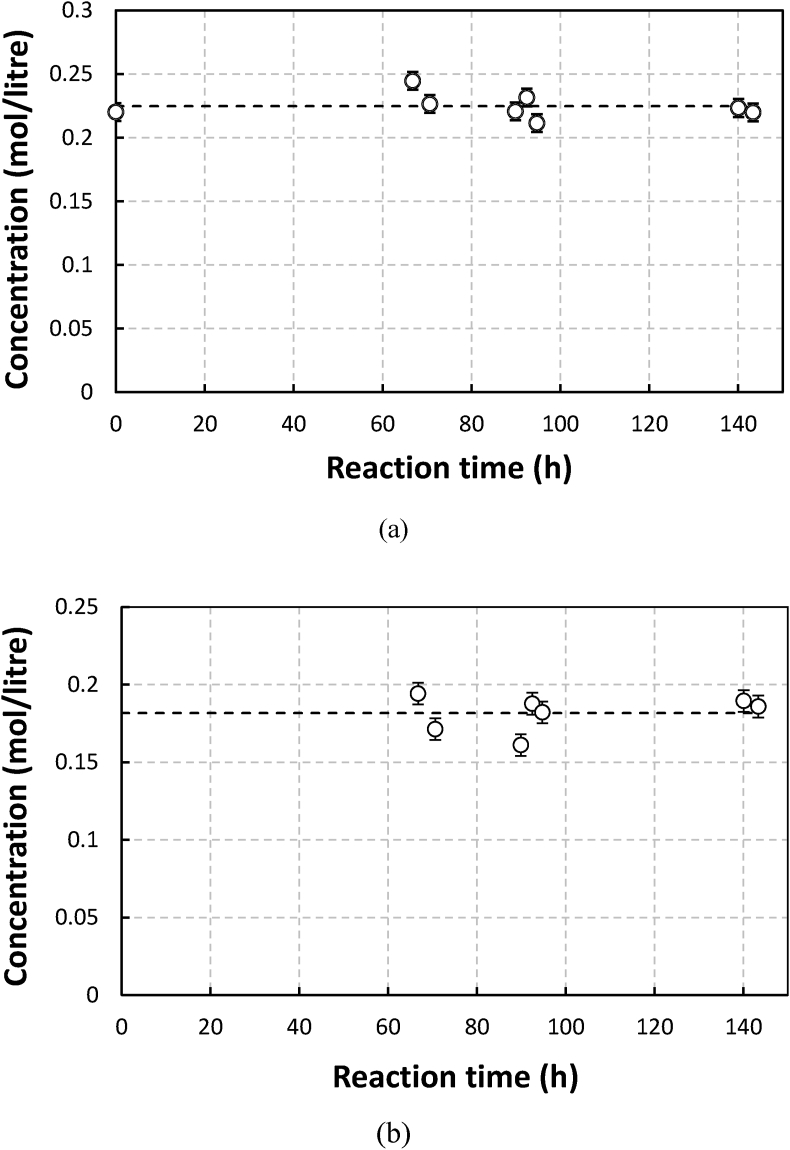
The stability of EDTA ligands in an aerated EDTA solution without sulfides at pH of 8: (a) without iron and (b) with iron (0.1 M).

**Figure 3:**
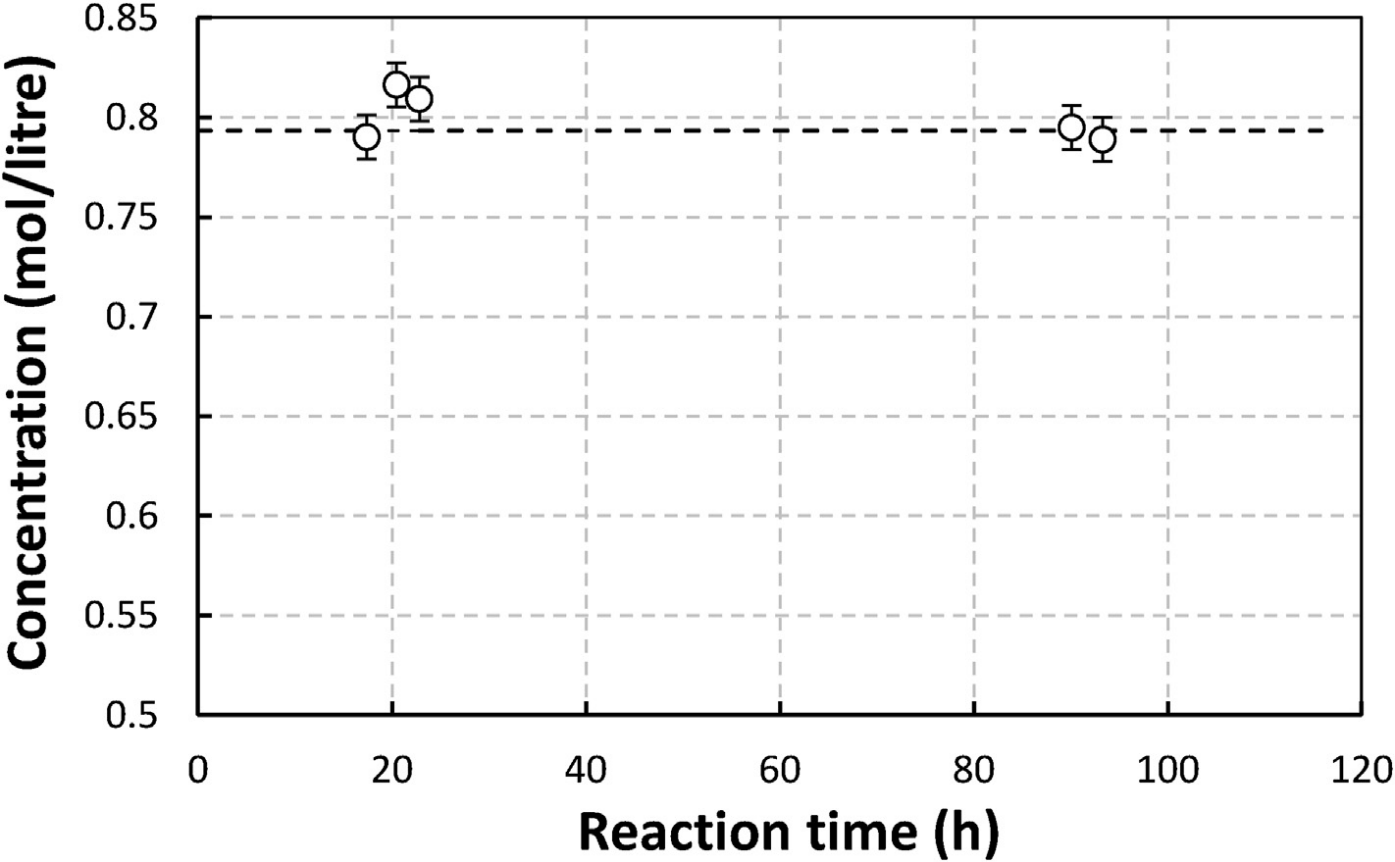
The stability of gluconate ligands in an aerated Manganese gluconate solution without sulfides at pH of 13.

The stability trends of EDTA and gluconate in the solution at pH of 8 and 13 are shown in Figure 2 and Figure 3, respectively.

As shown in Figures 1, 2, and 3, the results were in agreement with the observations made by Chen et al. [6]. With and without metals, in the absence of sulfide, the concentrations of NTA, EDTA, and gluconate tended to be constant throughout the observation time for 100–140 h. For solution with metals, this means that within the time range and operating conditions applied in the work, neither autocatalyzed oxidation nor Ruff degradation (despite the possible formation of H_2_O_2_ during Manganese (II) oxidation) was significant. The observations made by Piché about the relatively fast degradation of EDTA in Fe-EDTA solution without the presence of sulfide and aeration [7], was therefore interesting. In verifying the composition of degradation products, Piché found to 80% of this consisted of Fe(OH)_3_ while the remaining 20% was Fe Chelate adsorbed in the product. Based on our calculation using data from Stum and Morgen ie., 27.7 (log K_ML_), log K_M(OH)L_ of 33.8, and Fe(OH)_3_ solubility product of -log (K_So_) of 42, at pH 8, this could have still resulted in precipitation of Fe(OH)_3_. If the chelate to metal ratio (RCM) used had been around 1.5 or more, which is typical for Fe-EDTA, there might have been no iron hydroxide precipitation. Piché used an inline UV visible spectrophotometer for measuring chelate concentration. Samples taken from the reactor were directly pumped to the device. Therefore the actual chelate/iron molar ratio could be more than 1 most of the time due to iron precipitation. Hence in this case the spectrophotometer may only detect chelates attached to metal [3]. Free chelate concentration was not measured. This and the fact that the degradation was demonstrated to increase with pH, may be indications that the degradation in Piché could have been due to de-chelation rather than chelate destruction by oxidation. A similar explanation may perhaps be used for Sonmex degradation observation as well. In this case, even though a chelate to metal molar ratio of 1.5 was used, de-chelation could have taken place due to relatively high pH (9) of Fe EDTA and the quite high metal concentration. It is not clear whether, in this specific degradation experiment, the total ligand concentration including non-coordinated ones was measured by Sönmez like in his other experiments. If this was not so, it may become difficult to know if oxidative degradation took place. Sönmez also found that when the Fe (0.1 M) -EDTA (0.15 M) solution was excluded from oxygen and was sparged with CO_2_ at 60 °C then within 700 h concentration of EDTA went down by 25% of the initial concentration. Motekaitis reported the first order constants of iron (III) catalyzed oxidation of EDTA at temperatures between 102 °C to 143 °C for Fe(III) and EDTA concentrations of 0.01 M and 0.015 M, respectively in absence of oxygen at pH of 9.3. If the data could be extrapolated to 60 °C, then the predicted degradation rate would be much lower than that of Sönmez at CO_2_-sparged conditions. Experiments with higher initial concentration and longer reaction time need to be performed with manganese gluconate to be able to clarify this matter.

### 3.2 Degradation rate of gluconate, NTA, and EDTA chelates in the presence of sulfide

**Figure 4:**
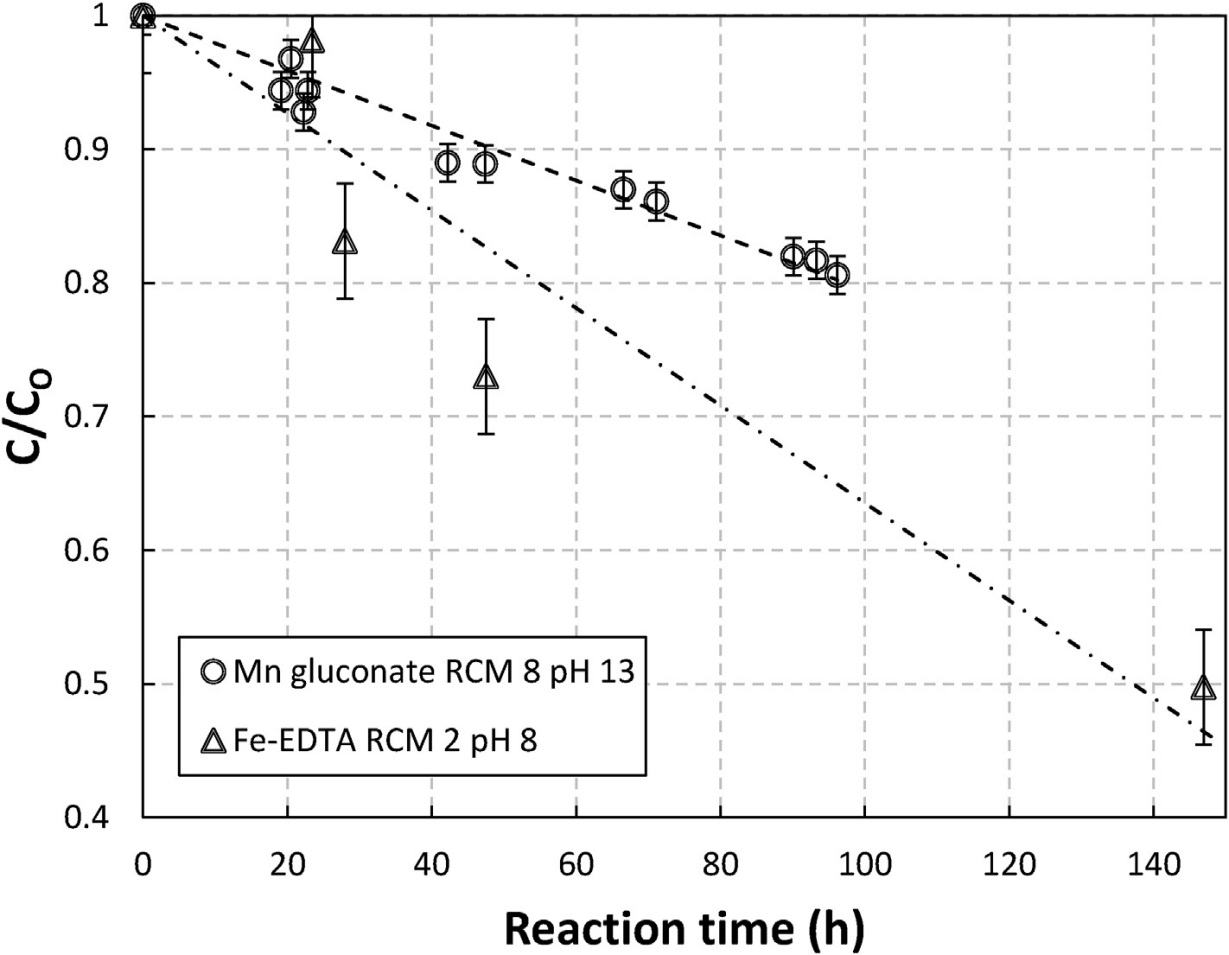
Relative Concentration (C/C_o_) profile of gluconate and EDTA chelates in aerated sulfide containing 0.1 M metal in metal chelate solutions as a function of time. Note: The initial ratio of chelate to metal on molar basis (RCM) and pH were 8 and 13 for manganese gluconate and for iron EDTA were 2 and 8, respectively.

**Figure 5:**
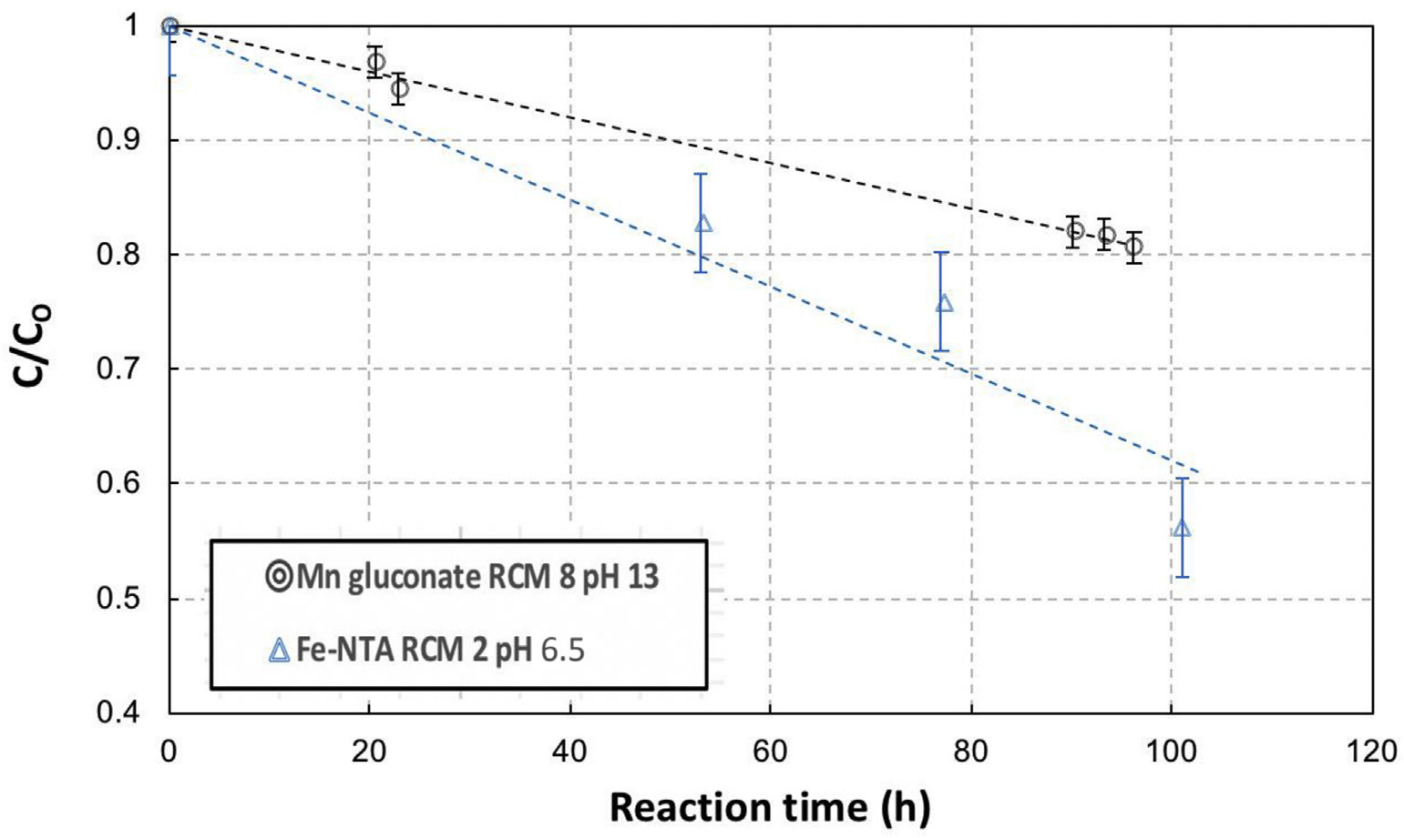
Relative Concentration (C/C_o_) profile of gluconate and NTA chelates in aerated sulfide containing 0.1 M metal in metal chelate solutions as a function of time. Note: The initial ratio of chelate to metal on molar basis (RCM) and pH were 8 and 13 for manganese gluconate and for iron NTA were 2 and 6.5, respectively.

Figures 4 and 5 show the degradation performance of gluconate, NTA, EDTA chelates against oxidative degradation with sulfide presence in solutions. They are presented in the changes of the ratio of actual chelate concentrations in solution respective to their initial chelate concentrations (herewith will be referred to as relative concentration [3]) as a function of reaction time. The solutions were aerated for a period of 100 h–140 h.

As the figures show, all of the chelates degraded quite rapidly. This might be explained by using the following simplified reactions R1, R2, R3, R4 mechanism [1].

(R1) $$M\left(II\right)L+O_{2}+2H_{2}\text{O}⇌M\left(III\right)L+H_{2}O_{2}+2OH^{-}$$

(R2) $$M\left(II\right)L+H_{2}O_{2}\;\to M\left(III\right)L+OH^{\cdot }+OH^{-}$$

(R3) $$L+OH^{\cdot }\;\to L^{*}$$

(R4) $$M\left(III\right)L+HS^{-}\;\to M\left(II\right)L+S$$

Reactions 1 illustrates that initially the metal (II) chelate complex (M(II)L) is oxidized by oxygen to form a metal (III) complex, producing hydrogen peroxide as a side product. This substance will then oxidize the metal (II) complex to produce a hydroxyl radical in Reaction 2, which is the Fenton reaction. In Reaction 3, the degradation of the chelates occurs because of the reaction between chelates (L) and hydroxyl radicals to form degradation products (L∗). The reaction sequence ends with Reaction 4, which shows that the metal (III) complex will be reduced by dissolved sulfides to form metal (II) complexes again.

According to reaction 1 when M(II)L runs out for example if there is no addition of the sulfide once they have been consumed, radical production stops as there would be no reaction R1 anymore. Therefore, the presence of sulfide all the time completes the redox cycle that produces hydroxyl radicals continuously which eventually may consume all of the chelates in the solution.

It is apparent from Figures 4 and 5 that gluconate degradation performance was superior to that of NTA and EDTA. Within around 100 h, the gluconate concentration has only decreased by about 20%, while the NTA by around 40%. For the 47-hour reaction time, EDTA concentration had dropped by 30% while gluconate had only decreased by about 11%. The fact that degradation of manganese took place only when sulfide was present and was negligible when sulfide was absent indicated that Fenton-like degradation was highly more dominant than Ruff degradation and autooxidation mechanism.

### 3.3 Effect of metals, pH, chelate to metal ratio on gluconate degradation

In order to obtain more understandings of gluconate degradation, experiments with a variation on types of metals, pH, and chelate to metal ratio were also carried out. The results are then discussed in comparison to literature studies on similar topics.

#### 3.3.1 Effect of metal types on gluconate degradation rate in dissolved sulfide oxidation system

**Figure 6:**
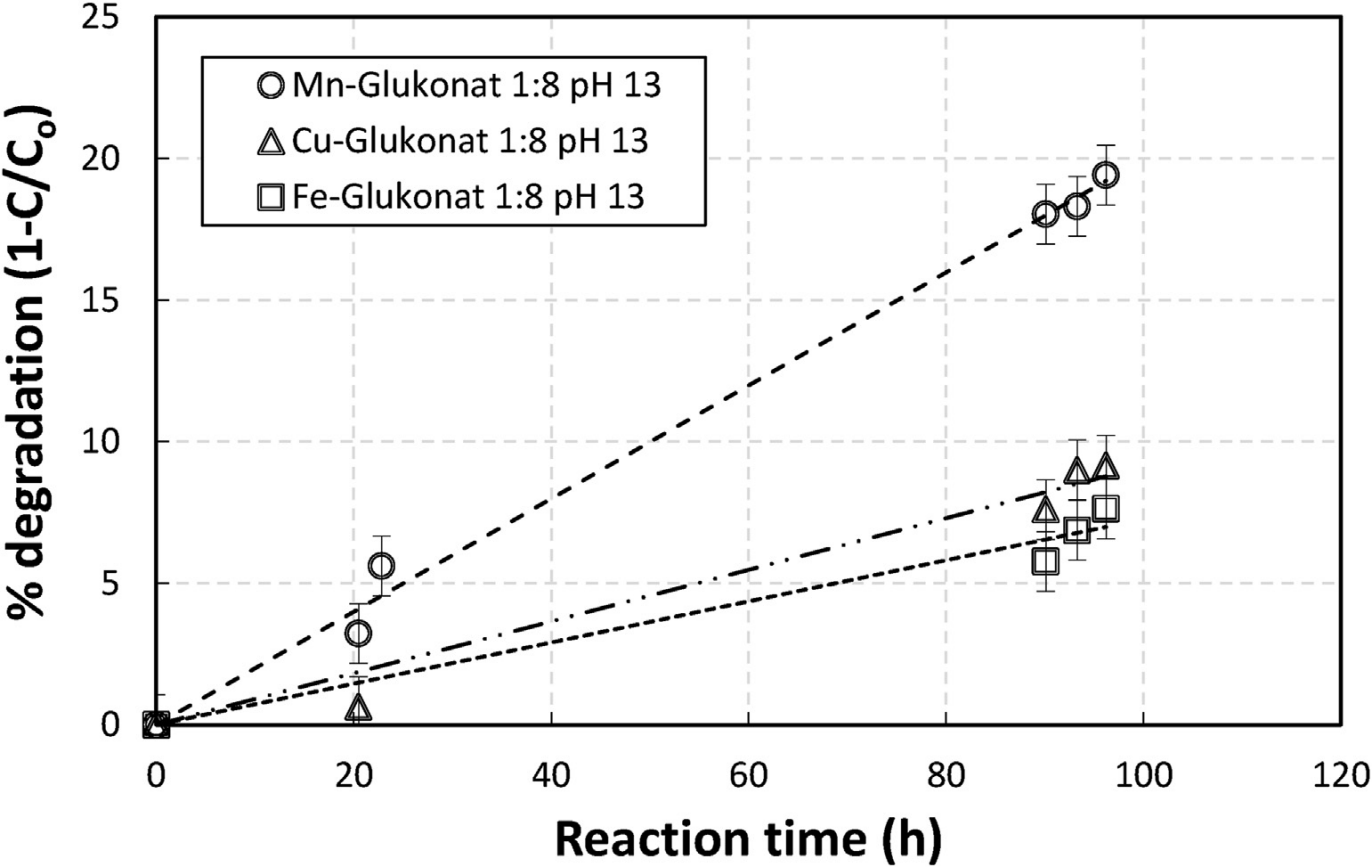
Extent of degradation (1-C/C_o_) profile of gluconate chelate in an aerated sulfide containing 0.1 M metal manganese, copper and iron gluconate solutions at pH of 13 and initial ratio of chelate to metal (RCM) of 8 as a function of time.

Figure 6 compares the degradation of gluconate when different metals were used. As Figure 6 demonstrates, in contrast to the system investigated by Cheton and Archibald [12] and by Valachova et.al. [11], manganese brought about a higher gluconate degradation rate than copper and iron. Within 100 h of reaction, gluconates that had been degraded by manganese, copper, and iron were ca 20%, 9%, and 7% respectively. This suggested that the lower degradation of manganese gluconate compared to Fe-NTA and Fe-EDTA observed in this work was not due to the use of manganese.

As mentioned before in the experiment Cheton and Archibald, Fe-EDTA was degraded more severely than Mn-EDTA. One of the possible reasons for the different results between their and our experiment was perhaps due to the different effect of gluconate and EDTA on the reduction potential of iron and manganese. In terms of thermodynamics, reaction 2 will take place more to the product side if the redox potential of H_2_O_2_/OH˙+ OHˉ at certain conditions is higher than the redox potential of M(III)L/M(II)L. Since data on the redox potential of various complexes are not always available, it is necessary to calculate them from the knowledge of the redox potential of the free metal and the complex stability constants [9, 16]. Thus, based on this method we estimated that the formal reduction potential of manganese gluconate at the prevailing experiment conditions (-0.45 V *vs* SHE) could be considerably less than iron gluconate (-0.27 V *vs* SHE). In other words, both manganese and iron gluconate can be oxidized by H_2_O_2_ to form a hydroxyl radical. Moreover, the driving force for the oxidation of manganese (II) oxidation was higher than that of iron (II) gluconate. Whereas the reduction potential of Manganese EDTA was rather high (0.82–0.85 V *vs* SHE at pH of *ca*. 7) [17] which is somewhat higher than the standard reduction potential of H_2_O_2_/OH∙ which is 0.8 V *vs* SHE [18]. Kinetically, the ability of gluconate to significantly improve the rate of manganese oxidation has been known in the literature. At neutral pH, Martin reported that the rate of free manganese (II) air oxidation was around a million times slower than oxidation of free iron (II) [19]. The free manganese oxidation only starts to take place at pH of 8 or higher, still at a much lower rate than that of iron. However, Bodini and Sawyer have shown that manganese (II) gluconate oxidation by oxygen can take place quite rapidly [9]. This has been confirmed as well in our currently unpublished work [20].

#### 3.3.2 Effect of pH on gluconate degradation rate in dissolved sulfide oxidation system

Chen et al. experiment show that in the presence of iron, NTA and EDTA degraded faster at lower pH. In manganese (II) gluconate oxidation as given by reaction 5, lower pH will shift the reaction equilibrium to the right.

(R5) $$2Mn\left(II\right){\left(GH_{3}\right)}_{2}\left(OH\right){\;}^{2-}+2O_{2}^{\cdot \cdot 2-}+4H^{+}⇋2Mn\left(III\right){\left(GH_{3}\right)}_{2}^{-2}+3H_{2}O_{2}$$

**Figure 7:**
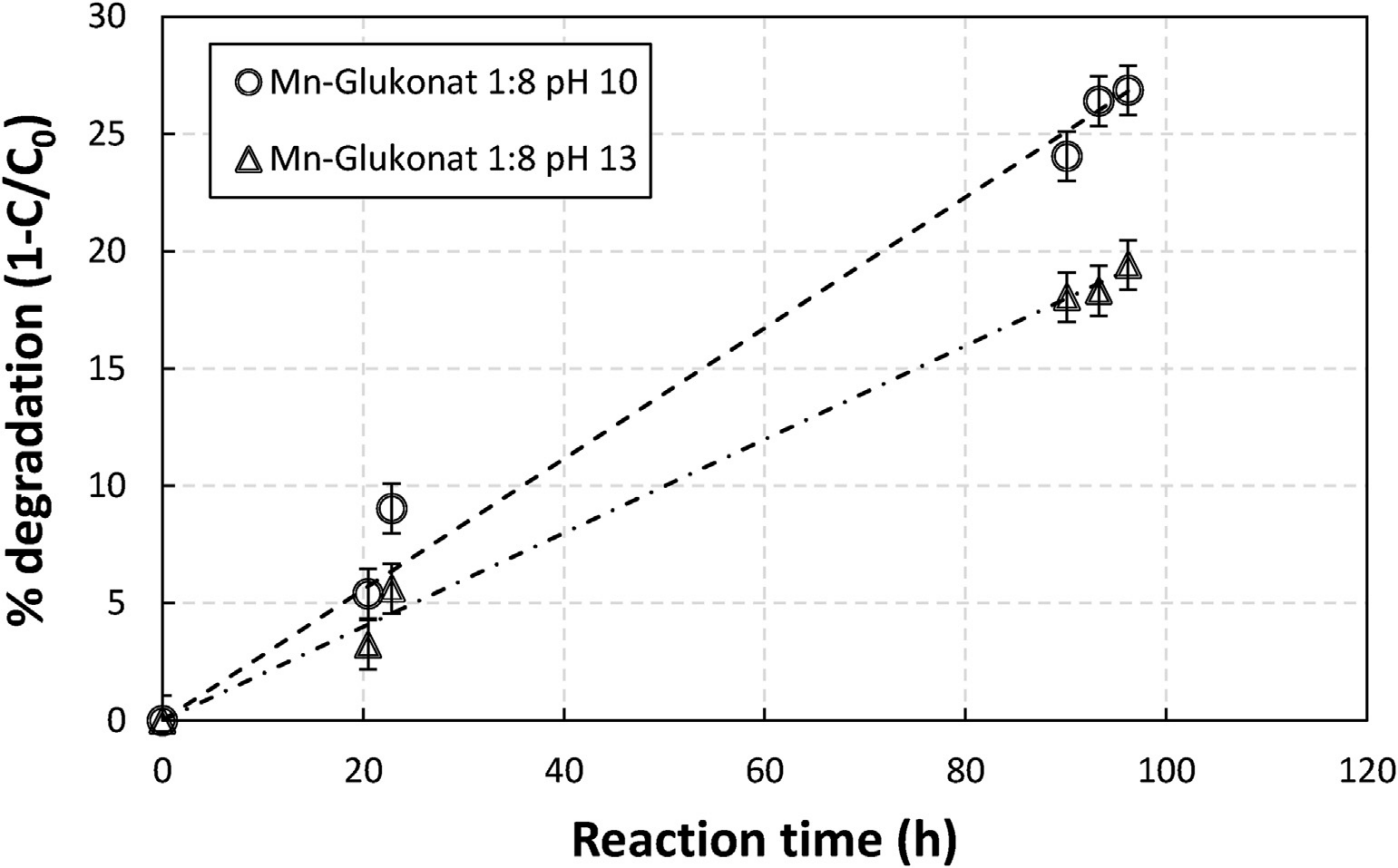
Effect of pH on the extent of degradation (1-C/C_o_) of gluconate in an aerated sulfide containing 0.1 M of Mn in manganese gluconate solutions as a function of time at initial chelate to metal ratio (RCM) of 8.

As shown in Figure 7, manganese gluconate degradation was also faster at a lower pH. Thus high pH application would be also favorable for manganese gluconate application in terms of chelate degradation. For iron EDTA and NTA, however, application at higher pH would have resulted in insolubility problems where metal-chelate bond might have weakened resulting in more iron precipitation.

#### 3.3.3 Effect of chelate to metal molar ratio on gluconate degradation rate in dissolved sulfide oxidation system

**Figure 8:**
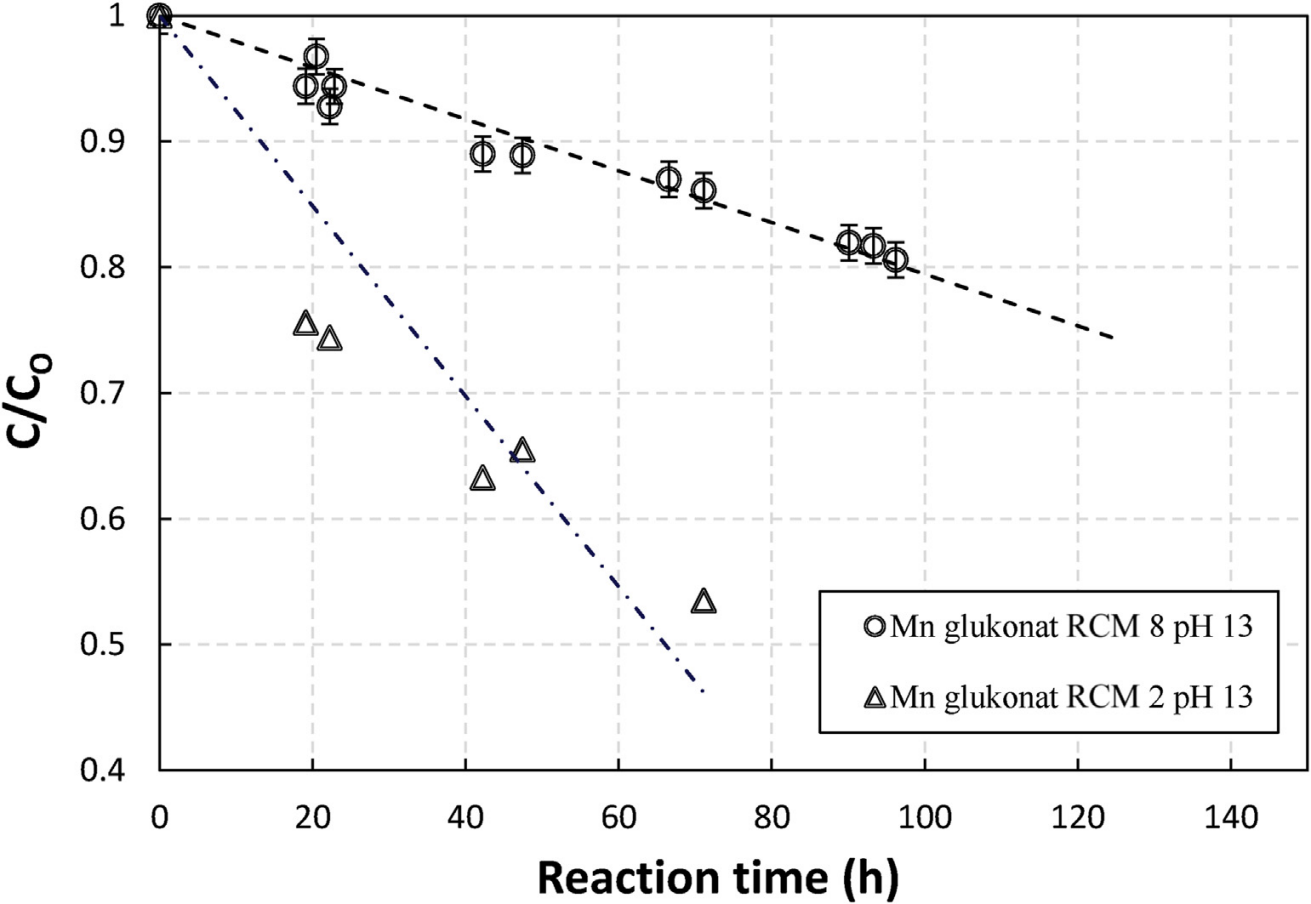
Effect of different chelate to metal molar ratios on relative concentrations (C/C_o_) of gluconate in an aerated sulfide containing 0.1 M of Mn in manganese gluconate solutions as a function of reaction time at pH 13. Note: RCM = chelate to metal ratio (in molar).

Figure 8 shows the changes in the extent of gluconate degradation as a function of reaction time and manganese gluconate solution at molar chelate to metal ratio (RCM) of 8 and 2. The latter is the stoichiometric RCM of Manganese (II) and (III) gluconate.

**Figure 9:**
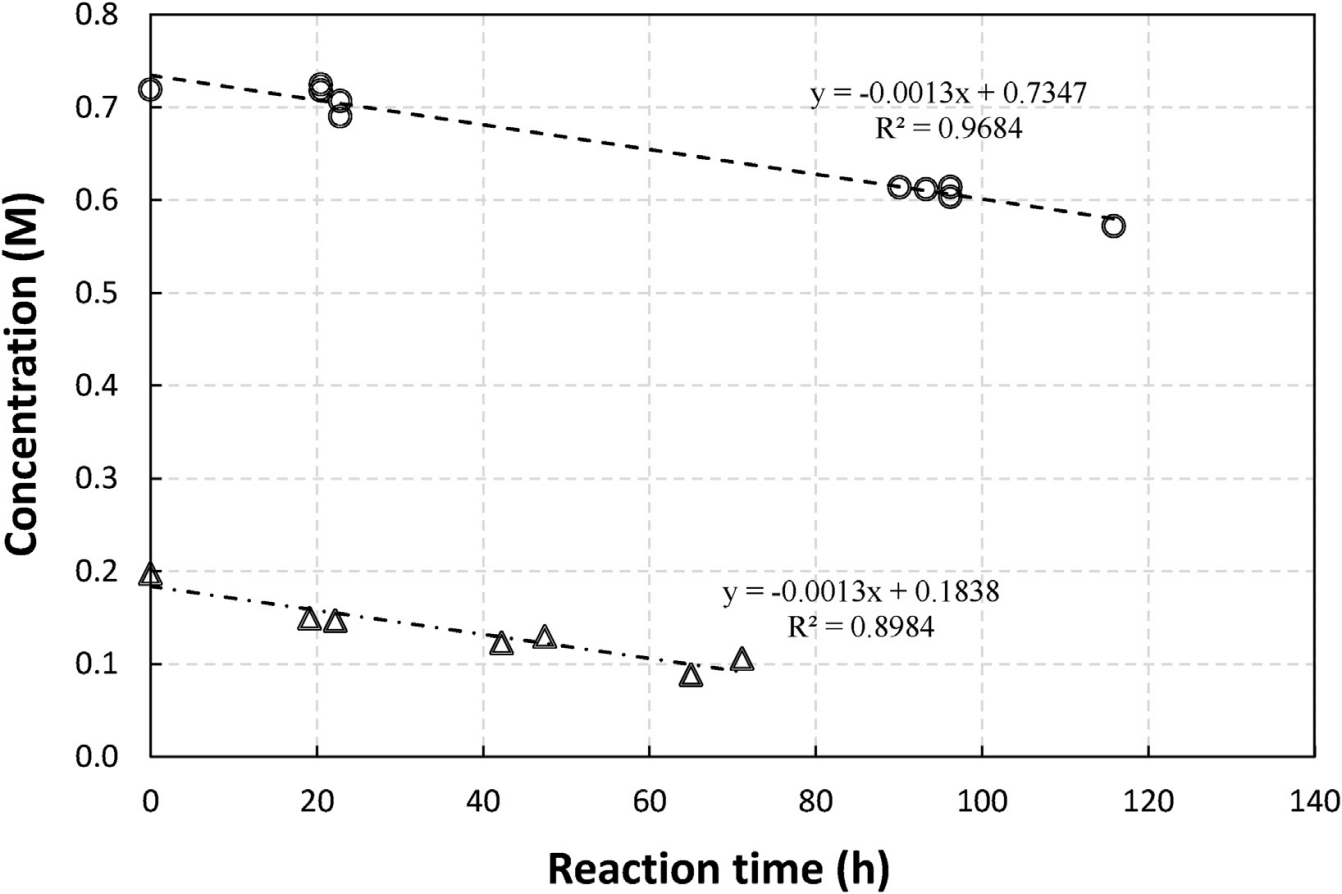
Concentrations of gluconate in an aerated sulfide containing 0.1 M of Mn in manganese gluconate solutions as a function of reaction time at pH 13 for RCM of 8 (circle) and RCM of 2 (triangle).

The apparent much faster degradation with the case of stoichiometric ratio may indicate that the chelates degraded were only those that coordinated with the metal. The same trend was observed with Fe NTA (not shown here). This observation is similar to those made by Sönmez who observed that non-coordinated EDTA chelate was not degraded in Fe-EDTA experiment [3]. If the degradation profile comparison is replotted using an absolute concentration of gluconate (as shown in Figure 9), it can be seen that at both RCM the gluconate reaction rate has the same rate constant, which supported that degradation may be taking place on coordinated chelate only.

## 4 Conclusions

The chelates in solutions of manganese gluconate, Fe-EDTA and Fe-NTA were shown to be quite stable in the aerated condition in the absence of sulfides. This might give an advantage for the industrial application as it would make operation easier and save shipping costs because chemical formulation could be sent in large quantities at once. In the presence of sulfides, the rate of gluconate degradation in the manganese gluconate system was considerably slower than the chelate degradation rate in the Fe-NTA and Fe-EDTA systems. Even though the molar ratio of chelate to metal is different, they are however in total price-wise more or less comparable. Experimental results including the effect of sulfides, pH, metal-chelate ratio, and the type of metal on degradation can be explained consistently using the degradation reaction mechanism by the generation of hydroxyl radicals as the main cause of degradation. The performance superiority of manganese gluconate in terms of degradation is mainly determined by the higher pH and the higher ratio of chelates to metal applied. With other advantages of gluconate, the better degradation of gluconate adds valuable points to its prospect to be used as a redox agent for LRSR applications.

## Declarations

### Author contribution statement

Andreas Widodo: Conceived and designed the experiments; Performed the experiments; Wrote the paper.

Yestria Yaswari: Performed the experiments.

Rina Mariyana: Performed the experiments; Analyzed and interpreted the data.

Aditya Farhan Arif: Analyzed and interpreted the data; Wrote the paper.

Tirto Prakoso: Analyzed and interpreted the data.

Tri Partono Adhi: Conceived and designed the experiments; Analyzed and interpreted the data.

Tatang Hernas Soerawidjaja: Conceived and designed the experiments.

Ronny Purwadi: Conceived and designed the experiments; Performed the experiments.

Antonius Indarto: Conceived and designed the experiments; Wrote the paper.

### Funding statement

The research was supported by PT. Rekayasa Industri, Engineering & Construction, Indonesia. This research is also partially funded by the Indonesian Ministry of Research, Technology and Higher Education under the World Class University (WCU) Program managed by Institut Teknologi Bandung.

### Data availability statement

Data included in article/supp. material/referenced in article.

### Declaration of interests statement

The authors declare no conflict of interest.

### Additional information

No additional information is available for this paper.
